# Supplementary material for: Improving the Pediatric Emergency Department Learning Experience: A Simulation-Based Orientation for Pediatric PGY 1 Residents
Source: MedEdPORTAL. 2020 Jun 30;16:10919. doi: 10.15766/mep_2374-8265.10919 (PMC7331952; doi:10.15766/mep_2374-8265.10919)
Supplement: Supplementary file 1 — Case 1 Status Asthmaticus.docxLab Handout Status Asthmaticus.docxCase 2 Sepsis.docxLab Handout Sepsis Case.docxCase Instructions for Facilitators.docxParticipant Surveys.docxDebriefing Tools and Teaching Points.docxCritical Actions Checklist.docx [file mep_2374-8265.10919-s001.zip › A. Case 1 Status Asthmaticus.docx]

| **Appendix A: MedEdPORTAL Simulation Case Template**    **SIMULATION CASE TITLE: Status Asthmaticus**    **AUTHORS: Nicholas Holzemer, MD, Sarah Tomlinson, MD** | |
| --- | --- |
| **PATIENT NAME: Nathan**    **PATIENT AGE: 10 years old**    **CHIEF COMPLAINT: Respiratory Distress** | |
|  | |
| **Brief narrative description of case** | A mother brings in her 10 year old son to the emergency room for respiratory distress. He has a history of asthma and has had several days of URI symptoms with now increasing wheeze and cough not responding well to home inhaler treatments. Anticipated interventions include DuoNeb administration, IV steroids, continuous albuterol, and IV magnesium administration. Laboratory work up to be overall unremarkable except for a respiratory alkalosis. After stabilization the child will be admitted to the ICU or moderate care. |
| **Primary Learning Objectives** | - Be able to perform a brief, focused initial assessment and exam for asthma - Learn the initial steps of stabilization for an asthma exacerbation and immediate treatment needed prior to staffing - Critically determine an evidence based work up including possible labs and imaging - Recognize partial response to treatment and when to move to second line agents - Learn when to get immediate help |
| **Critical Actions** | - Recognize respiratory distress secondary to status asthmaticus - Give three DuoNebs and reassess response - Give steroids PO or IV - Recognize lack of sufficient improvement and start continuous albuterol - Make child NPO - Obtain IV access, appropriate labs (VBG, BMP) - Discuss addition of IV magnesium with IV crystalloid bolus - Repeat assessment with stabilization and slow improvement and determine appropriate disposition to moderate care floor |
| **Learner Preparation** | The learner is working in the pediatric emergency room. Their patient arrives with chief complaint of respiratory distress. The nursing triage note read “10 yo male, with asthma history, coughing not responsive to home treatments. Tachypneic and labored, skin exam normal. No fevers.” (___) will be playing the role of the parent. The patient is ready in the room. |

| Initial Presentation | | | |
| --- | --- | --- | --- |
| **Initial vital signs** | Temperature 37.6 C, Pulse 115 bpm, BP 108/65 mm Hg, RR 32, SpO2 92% on room air. Weight 30 kg | | |
| **Overall Appearance** | Young boy, lying back against elevated bed. Appears somewhat afraid, frequent tight cough. He’s tachypneic and suprasternal retractions are visible over his shirt. Mother is present at bedside. | | |
| **Actors and roles in the room at case start** | Mother is present at bedside, will be played by senior facilitator or sole facilitator | | |
| **HPI** | *Mother offers*: Has had two days of runny nose, sore throat, nasal congestion. Last night he developed a nonproductive cough so started giving him his inhalers. When was not improving with treatment overnight came to the emergency room.    *Additional information when prompted*: She has noted some wheezing last night with some relief from albuterol inhaler. Last dose was 2 hours ago, 4 puffs via albuterol inhaler. Tolerating PO though somewhat less interested since last night, no vomiting. No fevers, chills, sweats, headaches, ear pain, abdominal pain, diarrhea, dysuria, urinary frequency, rashes. He attends school and several other children have been sick with cold like symptoms. He did have his influenza vaccine this year. Has not been taking his controller inhaler as prescribed. | | |
| **Past Medical/Surgical History** | **Medications** | **Allergies** | **Family History** |
| Moderate persistent asthma with 3 previous hospital admissions, 1 previous PICU admission, no intubations. Current baseline is occasional nighttime coughing, uses albuterol 1-2 times a week.    Seasonal allergic rhinitis | Beclometasone dipropionate inhaler  Albuterol inhaler 2-4 puffs q4h prn  Cetirizine daily | Ragweed | Mother with eczema and asthma  Father with seasonal allergic rhinitis  No siblings. |
| **Physical Examination** | | | |
| **General** | Young boy in **moderate respiratory distress, acknowledges your entry but speaking in one word sentences.** | | |
| **HEENT** | Pupils equal, round, and reactive. No conjunctival injection. Tympanic membranes translucent with normal light reflex. **Mild clear rhinorrhea, mild posterior pharyngeal erythema without exudates.** | | |
| **Neck** | Scattered anterior and posterior cervical lymphadenopathy. Normal jugular venous pressure. Supple with normal range of motion. | | |
| **Lungs** | **Intermittent tight cough. Occasional scattered faint wheezes but very poor aeration throughout. No rales or stridor. Suprasternal and subcostal retractions and belly breathing** | | |
| **Cardiovascular** | **Tachycardic,** normal S1, S2 with normal physiologic splitting. I/VI soft systolic ejection murmur at left upper sternal border. 2+ radial and dorsalis pedis pulses | | |
| **Abdomen** | Nontender, non-distended. No palpable organomegaly. Normal bowel sounds. | | |
| **Neurological** | Follows directions appropriately. Alert and oriented to person, place, time, and situation. Cranial nerves are intact and symmetric. Moving all extremities equally. | | |
| **Skin** | Normal turgor, warm and well perfused. No visible rashes. | | |
| **GU** | Deferred | | |
| **Psychiatric** | Following commands and answering questions appropriately. **Mildly anxious appearing.** | | |

| Instructor Notes - Changes and CASE Branch Points | | |
| --- | --- | --- |
| **Intervention / Time point** | **Change in Case** | **Additional Information** |
| 1st DuoNeb started | SpO2 saturations rise to 99% while on therapy  Heart rate increases 20 bpm  No change in lung exam yet |  |
| Nasal cannula placed, prior to DuoNebs given | Child becomes restless, taking off cannula. | Patient states: “Get this off me, I can’t breathe” |
| 2nd and 3rd DuoNeb given | Patient heart rate maintains ~140 bpm  Saturations maintain 99-100%  Lung exam with increased aeration and now diffuse wheezing, continued retractions |  |
| Participants recognize only marginal improvement with DuoNebs, start patient on continuous albuterol | Over time, retractions begin to resolve, wheezing less prominent but good aeration | Patient able to speak in full sentences now. Mom states “I think he’s starting to look a bit better” |
| If not started on continuous albuterol after last DuoNeb, OR patient not reassessed after DuoNebs given | Patient begins coughing more  Saturations decrease to 92%  Poor aeration now and less wheezing  Increased retractions | Mom states “He seems to be working a lot harder to breathe” |
| After starting on continuous albuterol, mom asks, “Can I give him something to eat?” | Learner should explain we keep children NPO while on continuous albuterol in the ED.  Will start maintenance fluids (NS or NS+K) to keep children hydrated once IV access obtained |  |
| IV access obtained and learner orders blood gas | 7.48/31/50, lactate 1.8 | Learner should be asked to interpret results as appropriate respiratory alkalosis |
| Learner requests CBC (not required) and BMP | WBC 12.4, Hgb 14.5, Plts 390  Na 141, K 3.7, Cl 108, HCO3 23, BUN 7, Cr 0.7, Gluc 68 | Baseline Cr 0.6 |
| Learner request chest x-ray (not required as non-focal exam) | Chest x-ray reported as symmetric hyperinflation, peribronchiolar thickening, no focal opacity |  |
| Learner request EKG (not required) | Verbally told EKG is sinus tachycardia without acute changes |  |
| Patient given magnesium sulfate (25-50 mg/kg) bolus and crystalloid 20 cc/kg IV bolus | Patient progressively shows decreased retractions, decreased wheezing.    IF NO crystalloid bolus is given, BP decreases to 88/60 and HR to 150 bpm. Patient is asymptomatic. | Mom states “He is starting to look much more comfortable”    IF NO bolus, mom asks “Why is his blood pressure so low” |
| Learner requests respiratory viral panel, procalcitonin, or CRP | Learner told these will not return while patient in emergency room, but have been collected |  |

**Ideal Scenario Flow**
Learners enter the patient room to find him in moderate respiratory distress. While performing a focused physical exam, they place the patient on bedside monitors. They recognize mild hypoxia for age and tachypnea. Focused history is obtained and DuoNeb treatments are initiated for presumed asthma exacerbation. Once treatment has begun, a more complete history and exam is obtained. On serial reassessments the patient’s lung exam shows improved aeration but continued increased significant work of breathing. The patient is then placed on continuous albuterol nebulization therapy at 15 mg/hr and held NPO. Learner decides IV over PO steroids based on the patient’s poor respiratory status. IV access is obtained with labs drawn at this time and patient is started on maintenance fluids and held NPO. Labs return with mild leukocytosis, normal basic metabolic panel, and venous blood gas with appropriate respiratory alkalosis and normal lactate. Chest x-ray (if obtained) shows symmetric hyperinflation with mild bronchial thickening, no focal opacities. On repeat assessment patient is very slowly improving so IV Magnesium sulfate and crystalloid bolus is given. After 2 hours of continuous albuterol therapy lung exam is significantly improved with diffuse wheezing but easier work of breathing and no retractions. Decision is made to admit patient to moderate care or the pediatric ICU.

**Anticipated Management Mistakes**

*Provide a list of management errors or difficulties that are commonly encountered when using this simulation case.*

1. Failure to recognize immediate need for bronchodilators: Learners may spend too much time focusing on obtaining a history or initiating a work up (e.g. chest x-ray, viral swabs, CBC/procal for pneumonia) and not initiate albuterol/DuoNeb treatments early. If this occurs the child should continue to worsen with increased tachypnea and decreasing saturations. If excess time passes and labs are obtained prior to any nebulized treatments given, would recommend altering blood gas to 7.40/45 showing he is no longer ventilating appropriately for his level of tachypnea.

2. Excessive lab and imaging ordered: We found several learners ordering an excessive amount of labs, such as CBC, CRP, procalcitonin without any other signs or symptoms of a bacterial pneumonia as well as chest x-rays without focal findings on exam. We used this as a learning point to discuss what labs will actually inform your management.

3. Unsure how to give steroids: Many learners debated how to give steroids and many delayed administration. This patient’s degree of respiratory distress makes him higher risk for oral intake and thus should remain NPO. We recommend discussing the risks and benefits of IV versus PO administration and appropriate dosing. Goal was to reinforce that they are equal in efficacy, and decision should be based on safety of administration.
