# Supplementary material for: Improving the Pediatric Emergency Department Learning Experience: A Simulation-Based Orientation for Pediatric PGY 1 Residents
Source: MedEdPORTAL. 2020 Jun 30;16:10919. doi: 10.15766/mep_2374-8265.10919 (PMC7331952; doi:10.15766/mep_2374-8265.10919)
Supplement: Supplementary file 1 — Case 1 Status Asthmaticus.docxLab Handout Status Asthmaticus.docxCase 2 Sepsis.docxLab Handout Sepsis Case.docxCase Instructions for Facilitators.docxParticipant Surveys.docxDebriefing Tools and Teaching Points.docxCritical Actions Checklist.docx [file mep_2374-8265.10919-s001.zip › B. Lab Handout Status Asthmaticus.docx]

**APPENDIX B – Lab Handout for Status Asthmaticus Case**

WBC 12.4

Hgb 14.5

Plts 390

141 | 108 | 7 /

3.7 | 23 | 0.7 \ 98

VBG 7.48 / 31 / 50 / 23 Lactate: 1.8
