# Supplementary material for: Improving the Pediatric Emergency Department Learning Experience: A Simulation-Based Orientation for Pediatric PGY 1 Residents
Source: MedEdPORTAL. 2020 Jun 30;16:10919. doi: 10.15766/mep_2374-8265.10919 (PMC7331952; doi:10.15766/mep_2374-8265.10919)
Supplement: Supplementary file 1 — Case 1 Status Asthmaticus.docxLab Handout Status Asthmaticus.docxCase 2 Sepsis.docxLab Handout Sepsis Case.docxCase Instructions for Facilitators.docxParticipant Surveys.docxDebriefing Tools and Teaching Points.docxCritical Actions Checklist.docx [file mep_2374-8265.10919-s001.zip › C. Case 2 Sepsis.docx]

| **Appendix C**  **MedEdPORTAL Simulation Case Template**    **SIMULATION CASE TITLE: Sepsis in setting of short-gut syndrome**    **AUTHORS: Nicholas Holzemer, MD, Sarah Tomlinson, MD** | |
| --- | --- |
| **PATIENT NAME: Leslie**    **PATIENT AGE: 4 years**    **CHIEF COMPLAINT: Fever** | |
|  | |
| **Brief narrative description of case** | A parent brings in their 4-year-old daughter with short gut syndrome and an indwelling central line to the emergency department for a fever without any localizing symptoms. On arrival the learner should recognize vitals and exam concerning for sepsis. Anticipated interventions include IV crystalloid fluid resuscitation, blood cultures followed by early administration of broad spectrum antibiotics, recognition of an AKI and lactic acidosis. Work should include serial focused assessments to gain more information while concurrently performing resuscitation. Once stable they will be admitted to the pediatric general floor. |
| **Primary Learning Objectives** | - Be able to perform a brief, focused initial assessment and exam on an unstable patient with suspected sepsis - Recognize tachycardia and hypotension as signs of sepsis in a young child - Learn how to monitor response to IV fluid resuscitation - Learn the components of the sepsis bundle - Learn when to get immediate help |
| **Critical Actions** | - Recognize concern for sepsis based on symptoms including tachycardia, fever, mild hypotension, altered mental status and skin mottling - Initiate early treatment with IV fluid resuscitation. - Obtain cultures and give broad spectrum antibiotics as early as possible. - Recognize need for additional fluid boluses - Obtain appropriate labs - Interpret a lactic acidosis and AKI can be consistent with sepsis. - Triage stabilized patient to a floor admission |
| **Learner Preparation** | The learner is working in the pediatric emergency room. Their patient arrives with chief complaint of fever. The nursing triage note read “4 yo female, with short gut with central line, 1 day fever. Respirations unlabored, skin exam normal. No recent travel.” (___) will be playing the role of the parent. The patient is ready in the room. |

| Initial Presentation | | | | |
| --- | --- | --- | --- | --- |
| **Initial vital signs** | | Temperature 39.4 C, pulse 160 bpm, blood pressure 86/55 mmHg, respiratory rate 30, SpO2 97% on room air, weight 12 kg. | | |
| **Overall Appearance** | | Young girl, lying in bed looking fatigued and ill. She doesn’t acknowledge you coming in. Parent is at bedside. | | |
| **Actors and roles in the room at case start** | | Parent is present at bedside, will be played by senior facilitator or sole facilitator | | |
| **HPI** | | *Parent offers:* Symptoms started last night with fatigue. This morning was noted to be febrile with temperature 101.1º F. Continued to feel tired and they rechecked the temperature a few hours later and it was 102.1º degrees. Had been feeling well prior to last night.    *Additional information when prompted*: They have noted poor appetite in the last 12 hours, but no nausea, abdominal pain, vomiting, or diarrhea. She has had no redness or drainage around her central line, no recent manipulations besides normal TPN delivery. No headaches, ear pain, rhinorrhea, cough, sore throat, dysuria, urinary frequency, rashes. She does not attend daycare/school. She is up to date on her vaccines. | | |
| **Past Medical/Surgical History** | | **Medications** | **Allergies** | **Family History** |
| - Short gut syndrome secondary to necrotizing enterocolitis and small bowel resection  - Premature birth at 28 weeks  - G-tube dependence  - TPN dependence  - Broviac central line placement | | Ranitidine BID  Pediasure tube feeds  TPN | None | Father with hypertension  Mother with preeclampsia  Healthy sister |
| **Physical Examination (only provide what is asked/evaluated)** | | | | |
| **General** | Young girl,  **ill appearing, tired, does not acknowledge your entry** | | | |
| **HEENT** | Pupils equal, round, and reactive. No conjunctival injection. Tympanic membranes translucent with normal light reflex. **Dry mucous membranes**. No rhinorrhea, posterior pharyngeal erythema, exudates. | | | |
| **Neck** | No cervical lymphadenopathy. Supple with normal range of motion. | | | |
| **Lungs** | Normal effort but **tachypneic**. No rales, stridor, wheezing. Good aeration. | | | |
| **Cardiovascular** | **Tachycardic**, normal S1, S2 with normal physiologic splitting. I/VI soft systolic vibratory murmur at left sternal border. **3+ radial and dorsalis pedis pulses** | | | |
| **Abdomen** | Nontender, non-distended. No palpable organomegaly. Normal bowel sounds. **G-tube in place without abnormal drainage, surrounding erythema, skin breakdown.** | | | |
| **Neurological** | **Minimally interactive, sleeping against parent. Wakes to voice or light touch.** Cranial nerves grossly intact. Moving all extremities equally. | | | |
| **Skin** | Normal turgor, **warm.** **Some mottling on the bilateral legs. Cap refill 3-4 seconds.** No visible rashes. **Broviac in place on right upper chest without abnormal drainage, surrounding erythema, skin breakdown.** | | | |
| **GU** | Deferred | | | |
| **Psychiatric** | **Minimally interactive** | | | |

| Instructor Notes - Changes and CASE Branch Points | | |
| --- | --- | --- |
| **Intervention / Time point** | **Change in Case** | **Additional Information** |
| *Obtain IV access* |  | *Should be done through Broviac* |
| *5 minutes into the case* | *BP begins decreasing if no IV fluid bolus has been given for hypotension* | *BP cuff recycles at 80/50* |
| *20 cc/kg IV fluid bolus given* | BP improves to 95/55 and heart rate slows by 10 bpm | *Minimal improvement in exam* |
| *Acetaminophen is given* | *Temperature improves to 37.5 C, but HR only decreases 5 bpm* |  |
| *VBG requested* | VBG: 7.29/33/50  Lactate 4.1  Glucose 90 |  |
| CBC, CMP requested | WBC 17.4, Hgb 11.2, Plts 250  Na 141, K 3.9, Cl 107, HCO3 15, BUN 15, Cr 0.9, Glucose 98  Prot 5.4, Alb 3.0, Alt 24, AST 30, AlkP 190, Bili 0.5 | Labs provided as hand out  Baseline creatinine 0.5  Baseline Hgb 11.0 |
| 5 minutes after repeat vitals obtained after the first bolus is given | If no second bolus is given BP returns to 86/55 and HR to 155 | Mom asks why blood pressure has gone down again |
| *2nd 20 cc/kg IV fluid bolus given* | BP improves to 98/60 and heart rate slows by 20 bpm. | *Child is now more alert, asking for food.* |
| 2nd VBG requested | VBG: 7.35/37/52  Lactate 1.9  Glucose 95 |  |
| Chest and abdominal radiograph requested | Impression is “Normal chest xray with broviac in proper placement at the right atrial - superior vena caval junction, unremarkable abdominal x-ray” |  |
| EKG requested | Reported as ”sinus tachycardia” |  |
| Learner requests respiratory viral panel, procalcitonin, or CRP | Learner told these will not return while patient in emergency room, but have been collected |  |

**Ideal Scenario Flow**

The learner enters the room to find the patient ill-appearing with parent at bedside. Upon initial set of vitals they recognize tachycardia, mild hypotension, and fever suggestive of sepsis. Focused physical exam shows no localizing signs of infection but is concerning for mottled appearance of the skin, altered mental status, poor capillary refill, but shows no signs of cardiac failure. Access is obtained through the patient’s Broviac, labs and cultures are drawn, and IV fluid resuscitation is started with 20 cc/kg IV crystalloid bolus. Having obtained cultures, broad spectrum antibiotic coverage is ordered such as vancomycin plus either cefepime+metronidazole or piperacillin/tazobactam. At this point additional history is gathered and full exam is completed with no localizing signs or symptoms. Blood gas returns with a lactic acidosis. On reassessment after the first bolus BP has improvement to 95/55 and heart rate is lower, but remains tachycardic. Temperature has also improved if acetaminophen has been given. The child is a little more alert but still has some mottling and delayed cap refill. A second bolus of IV crystalloid is given. Additional labs return with a leukocytosis, anion gap metabolic acidosis, mild acute kidney injury, normal hemoglobin, platelets, and hepatic function panel. If procalcitonin, c-reactive protein, or respiratory viral panel are ordered they do not return by the end of the session. Imaging, if obtained, shows a normal chest xray with broviac in proper placement at the right atrial - superior vena caval junction, unremarkable abdominal x-ray. EKG shows sinus tachycardia. After second IV fluid bolus is completed, heart rate improves to 120s, BP 98/68. On patient reassessment she is now interactive and cooperative with exam. A repeat blood gas, if obtained, shows improving lactic acidosis. Given stable vital signs and antibiotics initiated, decision is made to admit to the general care floor and the case ends.

**Anticipated Management Mistakes**

1. Failure to recognize sepsis: Some learners were concerned about dehydration and only started maintenance fluid or ascribing tachycardia only to fever, not recognizing the need for true fluid resuscitation. We found it helpful to further decrease the blood pressure and responsiveness of the patient to prompt recognition.

2. Failure to obtain cultures prior to antibiotics: Learners may forget to obtain cultures prior to antibiotic administration. Recommend providing additional prompting from the facilitator saying the nurse is about to get IV access and wondering what labs or cultures they want drawn.

3. Failure to treat with broad spectrum antibiotics: Some learners opted to treat with more narrow spectrum antibiotics such as ceftriaxone or ampicillin/sulbactam. Given the presence of a central line, short gut syndrome, and previous hospitalizations the child requires initial broad coverage. Recommend having the facilitator redirect them similar to an attending role, saying they do not believe the antibiotics are broad enough.
