# Supplementary material for: Improving the Pediatric Emergency Department Learning Experience: A Simulation-Based Orientation for Pediatric PGY 1 Residents
Source: MedEdPORTAL. 2020 Jun 30;16:10919. doi: 10.15766/mep_2374-8265.10919 (PMC7331952; doi:10.15766/mep_2374-8265.10919)
Supplement: Supplementary file 1 — Case 1 Status Asthmaticus.docxLab Handout Status Asthmaticus.docxCase 2 Sepsis.docxLab Handout Sepsis Case.docxCase Instructions for Facilitators.docxParticipant Surveys.docxDebriefing Tools and Teaching Points.docxCritical Actions Checklist.docx [file mep_2374-8265.10919-s001.zip › D. Lab Handout Sepsis Case.docx]

**Appendix D - Lab Handout for Sepsis Case**

**LABS**

VBG: 7.29 / 33 / 50 / 16, lactate 4.1, glucose 90

WBC 17.4

Hgb 11.2

Plts 250

CMP: 141 | 107 | 15 /

3.9 | 15 | 0.9 \ 98

Prot 5.4

Alb 3.0

AST 24

ALT 30

Alk phos 190

Tbili 0.5

Dbili 0.4
