# Supplementary material for: Improving the Pediatric Emergency Department Learning Experience: A Simulation-Based Orientation for Pediatric PGY 1 Residents
Source: MedEdPORTAL. 2020 Jun 30;16:10919. doi: 10.15766/mep_2374-8265.10919 (PMC7331952; doi:10.15766/mep_2374-8265.10919)
Supplement: Supplementary file 1 — Case 1 Status Asthmaticus.docxLab Handout Status Asthmaticus.docxCase 2 Sepsis.docxLab Handout Sepsis Case.docxCase Instructions for Facilitators.docxParticipant Surveys.docxDebriefing Tools and Teaching Points.docxCritical Actions Checklist.docx [file mep_2374-8265.10919-s001.zip › E. Case Instructions for Facilitators.docx]

**Pediatric ED Intern Orientation -- Resident/Fellow/Attending Instructions**

Thank you for participating in the orientation for our interns. We hope this can improve their confidence, efficiency, and management strategies in the ED throughout their training. We also hope this can provide good experience for you in simulation based teaching. The following is an introduction to how to use these simulation cases.

Cases should be presented in an oral format. The simulation equipment will include a high-fidelity mannequin capable of reproducing exam findings including breath and heart sounds and palpable pulses as well as a vitals board able to display telemetry leads, blood pressure, respiratory rate, oxygen saturation, and temperature. All of these can be actively manipulated during the cases and will help recreate the ED care experience as best as possible.

SESSION INTRODUCTION

Many students/residents may not have completed simulation based training prior this, so recommend start by introducing the basic tenets of simulation. Recommend discuss that the cases may be challenging and they may encounter new concepts outside their current scope of training. Everyone is doing their best and this is in no way an evaluative process. Simulation is meant to help learners discover areas of growth for themselves. We also recognize that this is an artificial, fictitious environment where things will likely not react with perfect life-like fidelity, but we aim to suspend disbelief to gain the most we can from each session. Finally, reinforce confidentiality, that this is a safe place to make mistakes and that what happens in the simulation room will stay there and not be reported to others including not to any medical school or residency supervisor.

AGENDA

If there are only 2-3 participants, one person will be the primary provider while the others will serve as additional residents in the department who can be consulted for help as well as provide feedback after the session. These roles can then rotate for the second case.

If 4 or more learners, recommend splitting into two groups, with each group having a team leader who will serve as primary provider while the others can be other on shift residents or nursing staff. The opposite group will be able to provide feedback and ask questions and then rotate in for the second case.

We encourage non-primary provider participants to practice using the ED context within the EMR using one of the practice patient environment. This will allow them to familiarize themselves with the order navigators as well as the documentation tab.

Depending on learner progress, we target each case lasting ~20 minutes including time for feedback and debriefing. This will allow additional time for 15-20 minutes for general expectations and ED work flow questions and discussion.

ROLES

When two facilitators are present, we encourage the junior member to lead the case and the more senior member to play the role of the parent. If only one facilitator is present they will play both roles. A simulation technician will be present to help run the simulation equipment unless one of the facilitators feel confident operating it themselves.

CASE FLOW

The vitals board should be turned off until cardiorespiratory monitoring is requested by the provider. Start each case with the chief complaint, patient age and gender, and then by providing the information in the nursing triage note. The case then begins and the provider can meet their patient.

Everything else should occur based on the prompting of the learners. This includes the reporting of initial vitals as well as general appearance of the child. The cases will summarize how much information should be offered to general questions of “what brings you to the emergency room?” The additional HPI can be provided by the parent when asked in a review of symptoms as the facilitator sees fit. Learners should examine mannequin as a real patient and should receive findings verbally from the case facilitator as they do so in case they cannot interpret the mechanical sounds. Only give them what they are asking for, for example don’t report a full exam back to them when they only ask “what do I see?,” only provide the general appearance exam. Our goal is that they perform a focused history and physical and initiate some work up and treatment before circling back for a more complete evaluation.

Regarding patient management, we will not require them to physically do any tasks that would otherwise be performed by nursing, such as breathing treatment administration. However, use the prompts of each case to make the mannequin and vitals respond as if the treatments are being given. Use closed loop communication to confirm that requested studies and therapies are being performed. We will also live in a fictional world where the RNs will not be following protocols and doing things prior to resident orders, e.g. no duo-nebs until they’ve ordered them. Ask for the name of the medications, dosages (it’s not expected they remember antibiotic dosing, but fluid boluses, albuterol, acetaminophen, steroids should all be reasonable expectations), route, and in what order (e.g. antibiotics immediately after cultures for the sepsis case, IV steroid for the asthmatic child that is too distressed to take PO).

Labs and imaging studies can be obtained with results of common tests (CBC, CMP, VBG, UA, CXR, EKG) returning shortly after request given time constraints. However, if other tests such as procalcitonin, CRP, viral panels, CTs, MRIs are ordered these will remain pending by the end of the case. Acting in the role of an attending as the facilitator, I ask the students to justify the labs and imaging they order and how it will inform their management. We also ask them to interpret the results aloud to the group.

If the learner is missing any critical actions or performing inappropriate interventions, the facilitator has two options. They can either allow the case to continue and alter the patient’s vitals and labs in response to this or the facilitator can act as the attending and step in to recommend alterations in the plan. Recommend favoring allowing the case to continue as possible unless time is getting short or it is clear that the learner is widely missing the diagnosis. Progressively directive prompting can also be used either from the “parent” facilitator or the lead facilitator, such as suggested in parts of the case script.

The rest of the case should be spent completing the physical exam, interview, workup and ongoing management of the patient to the point of being stable for admission. If this is not determined by the learner, the facilitator can stop the case by asking where the patient should be admitted now that they are stable.

DEBRIEFING

Recommend using the included debriefing guide for each case, document titled “Case Debriefs.” This includes questions to ask as well as salient points to make sure you cover in overall ED management and student reflection on their management.

EVALUATION

Included in the shared folder is an evaluation that you can distribute to the learners as well as your fellow facilitators if you see fit. Keeping record of these as you apply for academic positions can be helpful to show more formal feedback on your own teaching skills. These are entirely optional.
