# Supplementary material for: Improving the Pediatric Emergency Department Learning Experience: A Simulation-Based Orientation for Pediatric PGY 1 Residents
Source: MedEdPORTAL. 2020 Jun 30;16:10919. doi: 10.15766/mep_2374-8265.10919 (PMC7331952; doi:10.15766/mep_2374-8265.10919)
Supplement: Supplementary file 1 — Case 1 Status Asthmaticus.docxLab Handout Status Asthmaticus.docxCase 2 Sepsis.docxLab Handout Sepsis Case.docxCase Instructions for Facilitators.docxParticipant Surveys.docxDebriefing Tools and Teaching Points.docxCritical Actions Checklist.docx [file mep_2374-8265.10919-s001.zip › F. Participant Surveys.docx]

**Appendix F - Participant Surveys**

How confident do you feel in making care decisions independently in the pediatric emergency department?

1- not at all confident
2 -only slightly confident
3 -moderately confident
4 - mostly confident
5 - very confident

Based on your training thus far, how prepared do you feel to rapidly assess and stabilize unstable patients on arrival to the ED?

1- minimally prepared, I would leave these patients to more senior residents
2 - mildly prepared, but would try but with an attending/fellow nearby
3 - moderately well, prepared would feel comfortable with initial steps prior to getting attending/fellow input
4 - fully prepared to operate with minimal oversight

How would you rate your comfort level with regard to initiating treatment of a patient before staffing with a fellow or attending?

1- not at all comfortable
2 – slightly comfortable
3 – moderately comfortable
4 – mostly comfortable
5 – very comfortable

Did you find the case-based orientation didactic helpful in improving your care in the pediatric Emergency Department?

Not at all
Somewhat
Moderately
Very
