# Supplementary material for: Improving the Pediatric Emergency Department Learning Experience: A Simulation-Based Orientation for Pediatric PGY 1 Residents
Source: MedEdPORTAL. 2020 Jun 30;16:10919. doi: 10.15766/mep_2374-8265.10919 (PMC7331952; doi:10.15766/mep_2374-8265.10919)
Supplement: Supplementary file 1 — Case 1 Status Asthmaticus.docxLab Handout Status Asthmaticus.docxCase 2 Sepsis.docxLab Handout Sepsis Case.docxCase Instructions for Facilitators.docxParticipant Surveys.docxDebriefing Tools and Teaching Points.docxCritical Actions Checklist.docx [file mep_2374-8265.10919-s001.zip › G. Debriefing Tools and Teaching Points.docx]

**Case Debriefing Tools**

*Case 1: Asthma Exacerbation*

Consider using the following questions to help guide the debriefing session for the interns. Use of open-ended questions will help generate discussion.

Self Reflection

1. How did it feel when you were taking care of the patient?
2. What do you think you did well during this case?
3. What did you find challenging about this case?
4. At what point in your evaluation would you want to get the fellow and/or the attending involved?
5. If you read this triage note before seeing the patient, would you feel comfortable seeing this patient or would you wait to see if a senior resident would pick this patient up? Why is that?

Case Specific Medical Knowledge/Teaching Points

1. Frequent reassessment with bronchodilator administration: Make sure you are reassessing your patients receiving bronchodilators often. You should feel comfortable starting bronchodilators (Duonebs, albuterol) on a known asthmatic who presents with symptoms of an asthma exacerbation.
2. Early administration of steroids: Steroids administered early in a patient’s course improves their outcomes. These can be given orally (prednisone, prednisolone, dexamethasone), IM (dexamethasone), or IV (methylprednisolone, dexamethasone). Consider parenteral administration if patient is in severe respiratory distress.
3. When to obtain labs/imaging: Pure asthma exacerbations often do not need routine labs or chest x-ray. If you are placing a patient on continuous albuterol then an IV should be placed for IV fluids and perhaps IV magnesium or IV steroids. Labs should be obtained to follow potassium. Consider a blood gas if the patient appears to be decompensating or not responding to treatment as expected.
4. When to get help: When you are uncomfortable or not sure what to do next, if the patient appears to be tiring out, if the blood gas is concerning (particularly if there is a normal or elevated pCO2 despite the patient being tachypneic), or if the patient is having worsening hypoxia.

*Case 2: Pediatric Sepsis*

Self Reflection

1. How did it feel when you were taking care of the patient?
2. What do you think you did well during this case?
3. What did you find challenging about this case?
4. At what point in your evaluation would you want to get the fellow and/or the attending involved?
5. If you read this triage note before seeing the patient, would you feel comfortable seeing this patient or would you wait to see if a senior resident would pick this patient up? Why is that?

Case Specific Medical Knowledge/Teaching Points

1. Early recognition of signs and symptoms of sepsis: Children can be susceptible to delayed diagnosis of sepsis because they have very robust cardiovascular responses, and often you don’t see hypotension until late in the course. Additionally, toddlers and young children interact differently than adults, so altered mentation may be misinterpreted as disinterest, sleepiness (i.e., normal naptime), or fussiness.
2. Prompt management of the patient with suspected sepsis: Obtain cultures right away so that you can start antibiotics early. Antibiotics should ideally be started after cultures are obtained but treatment should not be unnecessarily delayed. Start fluids early and administer them quickly. Boluses should be given promptly, consider push-pull method, pressure bag, or rapid infuser to give bolus more quickly.
3. Determining responsiveness to fluid administration: You should expect to see improvements in heart rate, blood pressure, and mental status of the patient that is responding well to fluids. Patients should receive 60 ml/kg in IVF boluses before advancing to other medications for blood pressure support such as vasoactive infusions (epinephrine, norepinephrine, dopamine, etc) or stress-dose steroids.
4. EMR tools for sepsis management: Some institutions may have a sepsis bundle or sepsis order sets from which to choose labs, antibiotics, fluids, pressors, etc. They may also have a sepsis screen performed by triage or intake nursing that alerts the physicians to any patients that have risk factors for sepsis (based on their physical exam findings, vital signs, and medical history).
5. When to get help: Notify fellow/attending early when you are concerned that a patient has sepsis. Additionally, if the patient is not responding to fluid boluses, if there is worsening clinical status, or any delays in management (i.e., unable to get labs, IV access, etc).

*General ED Patient Care Teaching Points:*

1. Stress the importance of a focused history and physical exam to rule out life threatening causes. If you recognize that your patient is in distress, take a VERY focused history while obtaining a brief physical exam and then quickly initiate treatment (sometimes treatment can be initiated even before history is obtained depending on your exam findings). Consider use of the SAMPLE mnemonic for obtaining a quick history.
2. In general, if a patient appears sick or you are worried about someone, do not hesitate to excuse yourself from the room and have a fellow or attending come into the room with you.
3. Patients are triaged in the Emergency Department based on the severity of their chief complaint, abnormalities of their vital signs, any underlying medical conditions, and the expectations of how many resources they will need as part of their care. Patients such as those in the case scenarios would be assigned a higher triage category (indicating higher acuity), thus prompting a provider to see them more urgently than a lower acuity patient. Patients thus are seen in an order that sorts them based on triage category and time waiting to be seen. Most institutions will have an EMR that will automatically sort patients to make it easier for you to identify which patient needs to be seen next.
4. In the Emergency Department we try to quickly identify which patients are “sick” vs “not sick” -- which translates to “which patients are in need of resuscitation or immediate care” vs “which patients are stable enough to wait longer to be seen.” There are some things that may tip you off that a child is “sick” and needs your immediate attention. If there are significant vital sign abnormalities, this may be a sign that a child is significantly ill. If you walk into a room and a child is listless, doesn’t regard his/her parents, and/or is not interactive, these may be signs that a child is “sick” and needs immediate medical attention. In general, if a patient appears sick or you are worried about someone, do not hesitate to excuse yourself from the room and have a fellow or attending come into the room with you.
