# Supplementary material for: Improving the Pediatric Emergency Department Learning Experience: A Simulation-Based Orientation for Pediatric PGY 1 Residents
Source: MedEdPORTAL. 2020 Jun 30;16:10919. doi: 10.15766/mep_2374-8265.10919 (PMC7331952; doi:10.15766/mep_2374-8265.10919)
Supplement: Supplementary file 1 — Case 1 Status Asthmaticus.docxLab Handout Status Asthmaticus.docxCase 2 Sepsis.docxLab Handout Sepsis Case.docxCase Instructions for Facilitators.docxParticipant Surveys.docxDebriefing Tools and Teaching Points.docxCritical Actions Checklist.docx [file mep_2374-8265.10919-s001.zip › H. Critical Actions Checklist.docx]

**Case 1 - Asthma Exacerbation**

| Critical Action | Meets Expecations | Partially Complete/ With Prompting | Below Expectations | Comments |
| --- | --- | --- | --- | --- |
| Recognize respiratory distress secondary to status asthmaticus |  |  |  |  |
| Give three DuoNebs and reassess response |  |  |  |  |
| Give steroids PO or IV |  |  |  |  |
| Recognize lack of sufficient improvement and start continuous albuterol |  |  |  |  |
| Make child NPO |  |  |  |  |
| Obtain IV access, appropriate labs (VBG, BMP) |  |  |  |  |
| Discuss addition of IV magnesium with IV crystalloid bolus |  |  |  |  |
| Repeat assessment with stabilization and slow improvement and determine appropriate disposition to moderate care floor |  |  |  |  |

**Case 2 - Sepsis**

| Critical Action | Meets Expecations | Partially Complete/ With Prompting | Below Expectations | Comments |
| --- | --- | --- | --- | --- |
| Recognize concern for sepsis based on symptoms including tachycardia, fever, mild hypotension, altered mental status and skin mottling |  |  |  |  |
| Initiate early treatment with IV fluid resuscitation. |  |  |  |  |
| Obtain cultures and give broad spectrum antibiotics as early as possible. |  |  |  |  |
| Recognize need for additional fluid boluses |  |  |  |  |
| Obtain appropriate labs |  |  |  |  |
| Interpret a lactic acidosis and AKI can be consistent with sepsis. |  |  |  |  |
| Triage stabilized patient to a floor admission |  |  |  |  |
