# Supplementary material for: Improving the Pediatric Emergency Department Learning Experience: A Simulation-Based Orientation for Pediatric PGY 1 Residents
Source: MedEdPORTAL. 2020 Jun 30;16:10919. doi: 10.15766/mep_2374-8265.10919 (PMC7331952; doi:10.15766/mep_2374-8265.10919)
Supplement: Supplementary file 1 — Case 1 Status Asthmaticus.docxLab Handout Status Asthmaticus.docxCase 2 Sepsis.docxLab Handout Sepsis Case.docxCase Instructions for Facilitators.docxParticipant Surveys.docxDebriefing Tools and Teaching Points.docxCritical Actions Checklist.docx [file mep_2374-8265.10919-s001.zip › MEP-2019-0220_ESR_Table 2.FORMATTED.docx]

**Table 2.** PGY1 Resident Survey Responses Following The Second Year of Orientation Sessions

| **Questions** | **Pre-orientation**  **(n=10)** | **Post-rotation (n=11)** | **Mean difference** | ***p*** |
| --- | --- | --- | --- | --- |
| Confidence in independent decision making^a^ | 1.9 ± 0.6 | 2.6 ± 0.7 | +0.7 | 0.007* |
| Preparedness to rapidly assess and stabilize unstable patients^b^ | 1.6 ± 0.5 | 2.2 ± 0.7 | +0.6 | 0.01* |
| Comfort initiating treatment of a patient before staffing^a^ | 1.7 ± 0.6 | 2.4 ± 0.9 | +0.8 | 0.006* |

Survey answers given in Likert scales ranging ^a^[1 (lowest) - 5 (highest)], ^b^[1 (lowest) - 4 (highest)]

Each individual’s responses were paired between surveys for analysis. Results displayed as mean ± SD. Paired t-tests used for significance testing comparing pre-orientation and post-rotation scores.

*denotes significance of *p* < 0.05.
