# Supplementary material for: Improving the Pediatric Emergency Department Learning Experience: A Simulation-Based Orientation for Pediatric PGY 1 Residents
Source: MedEdPORTAL. 2020 Jun 30;16:10919. doi: 10.15766/mep_2374-8265.10919 (PMC7331952; doi:10.15766/mep_2374-8265.10919)
Supplement: Supplementary file 1 — Case 1 Status Asthmaticus.docxLab Handout Status Asthmaticus.docxCase 2 Sepsis.docxLab Handout Sepsis Case.docxCase Instructions for Facilitators.docxParticipant Surveys.docxDebriefing Tools and Teaching Points.docxCritical Actions Checklist.docx [file mep_2374-8265.10919-s001.zip › _Educational Summary Report_10919.pdf]

MedEdPORTAL® The AAMC Journal of Teaching and Learning Resources

Original Publication Open Access

# Improving the Pediatric Emergency Department Learning Experience: A Simulation-Based Orientation for Pediatric PGY 1 Residents

Nicholas F. Holzemer, MD*, Elaine S. Pomeranz, MD, Sarah Tomlinson, MD

*Corresponding Author: nholzemer@gmail.com

## Abstract

**Introduction:** Rotations in the pediatric emergency department (PED) may expose residents to very few critically ill patients. In our previous work, interns at our institution showed low self-confidence in decision-making and preparedness to stabilize acutely ill patients. In order to improve this, we designed a new, peer-led, simulation-based orientation to the PED rotation for interns focusing on workflow and decision-making. The cases presented learners with practical and generalizable challenges, such as ordering initial labs and medications and defining the ultimate disposition for the patient. **Methods:** This orientation curriculum was designed for first-year residents using high-fidelity simulation mannequins. In the first of two cases, learners managed a 10-year-old boy presenting with status asthmaticus who required continuous albuterol and parenteral magnesium to achieve stability for admission. In the second case, a 4-year-old girl with short gut syndrome and an indwelling central line presented with fever, was found to be septic, but responded well to fluid resuscitation and antibiotic therapy. **Results:** Over 2 years of implementation, 39 residents participated. Pre- and postintervention Likert-based survey evaluations showed significant increases in confidence in decision-making and preparedness to stabilize acutely ill children that were not seen in a control group during the pilot year. A subsequent class-wide implementation showed similar significant improvements, as well as increased comfort initiating treatment prior to staffing. **Discussion:** Using simulation mannequins in a case-based orientation can improve PGY 1 residents' self-confidence and sense of preparedness during their first rotation in the PED.

**Keywords**
Orientation, Pediatric Emergency Department, Pediatric Residents, Status Asthmaticus, Sepsis, Emergency Room Rotation, Simulation, Emergency Medicine, Pediatrics, Case-Based Learning

## Educational Objectives

By the end of this activity, learners will be able to:

1. Begin the approach to managing an undifferentiated patient in an emergent care setting.
2. Demonstrate the initial stabilization of a patient with respiratory distress and a patient with hypotension and tachycardia.
3. Describe the signs that a patient needs acute attention, as well as when to notify a fellow or attending for more help.
4. Gain confidence in managing an acutely ill child presenting to the emergency department.

**Citation:**
Holzemer NF, Pomeranz ES, Tomlinson S. Improving the pediatric emergency department learning experience: a simulation-based orientation for pediatric PGY 1 residents. *MedEdPORTAL*. 2020;16:10919. https://doi.org/10.15766/mep_2374-8265.10919

## Introduction

Previous evaluations have shown that rotations in the pediatric emergency department (PED) may expose residents to very few critically ill patients.[1,2] However, the recognition and care of these patients are important components of pediatric resident education as well as both inpatient and outpatient pediatric practice. Given this common disconnect, we sought to ascertain gaps in our own program and implement a new curriculum based on these findings.

In order to assess the status of residents' confidence in their independent decision-making and in their ability to stabilize critically ill children in the PED, an online survey was distributed to pediatrics and internal medicine-pediatrics (MP) residents in the tenth month of the academic year. Using a 5-point Likert scale, residents were asked to rate their level of self-confidence in independent decision-making and stabilization of unstable patients generally, as well as to provide specific examples of emergency department (ED) cases.

Copyright © 2020 Holzemer et al. This is an open-access publication distributed under the terms of the Creative Commons Attribution license.

Fifty-one residents completed the survey, including 16 interns (50% of the class). A majority of pediatric and MP interns and second-year residents near the end of the academic year felt no more than moderately confident in their independent decision-making in the ED as well as in their ability to stabilize unstable patients on arrival without significant oversight from an attending. By third year, only around half were confident about patient stabilization. Looking specifically at the intern class, 0% felt mostly or very confident in their decision-making, 65% felt moderately confident, and 35% felt only mildly confident. Regarding sense of preparedness to stabilize unstable patients on arrival without significant oversight from an attending, 59% of interns reported that they felt minimally prepared, and 41% reported being mildly prepared.

We aimed to improve these results by creating a new PED orientation. The preexisting orientation included a welcome email and a video tour of the PED prior to starting the rotation, followed by ED-focused didactic lectures asynchronously spread throughout the academic year as part of the daily resident lecture series. In the above survey, 60% of interns felt their orientation to the PED was inadequate, and 88% believed an in-person simulation-based session would be helpful. Additionally, 75% of interns said they would be willing to attend a session outside their usual PED shifts.

We chose a simulation-based format for our new orientation curriculum based on our residents' interest in a simulation-based orientation and also on previous work supporting the use of simulation to improve training. Simulation has been shown to improve test scores in surgical residents[3] and to reduce training gaps in pediatric emergency medicine (PEM) fellows.[4] Additionally, many studies report that simulation-based curricula have helped improve self-confidence in surgical residents,[5] obstetrics residents,[6] and pediatric critical care fellows.[7]

In choosing relevant topics, our findings showed that most interns did not feel confident performing even the initial steps of stabilization of asthma exacerbations (50%) or severe sepsis (69%). These were therefore chosen as higher-yield topics because they were also common presentations (respiratory distress, fever) with generalizable decision-making and management skills required.

There are numerous excellent simulation-based cases available in *MedEdPORTAL* teaching management of common pediatric emergencies, including status asthmaticus,[8] septic shock,[9] a four-case series on pediatric resuscitation,[10] and an instructive simulation of shock specifically meant for medical students.[11] There are also a few simulation curricula designed for orientations in other specialties, such as emergency medicine interns,[12] but none for orientations to the PED.

The focus of our curriculum was specifically to orient residents to workflow and decision-making within the PED to improve their self-confidence and sense of preparedness for patient care. While the details of the medical management were important, the cases were also meant to present learners with practical and generalizable challenges, such as decisions as to where patients should be managed (standard ED room vs. resuscitation bay), when more senior providers should get involved, and what workup and treatment an early trainee would be expected to accomplish prior to staffing with a fellow or attending. By framing the cases this way, we hoped to improve resident self-confidence, efficiency, and efficacy as ED providers from their very first experiences in the department. This curriculum was originally developed for pediatric and MP PGY 1 residents; however, it can be adapted for other trainees who care for patients in the ED setting.

## Methods

### Development

The previous relevant pediatric residency curriculum included didactic lectures on PEM topics asynchronously spread throughout the academic year. In addition, when residents started the ED rotation (generally 2-4 weeks in length), they received a welcome email detailing general rotation expectations and scheduling and a video tour of the department.

We wrote and developed the high-fidelity patient simulation cases. The simulation session was designed to serve as an adjunct to the current training curriculum.

As each intern's experience level could differ based on timing within the academic year, we provided two review articles on the content topics to be read prior to the session as needed: Jones et al.[13] and Mendelson.[14] We provided the facilitators with these articles, a handout describing the agenda and how to employ the cases (Appendix E), and the overall goals of the PEM rotation. We expected the facilitators to have knowledge of the cases and the topics addressed.

### Equipment/Environment

The orientation took place in either the resuscitation bay or a regular patient room within our PED, pending availability. We used a Gaumard pediatric simulation HAL 5-year-old mannequin in a stretcher as well as an electronic vitals board displaying heart rate, telemetry tracing, oxygen saturation, respiratory

Copyright © 2020 Holzemer et al. This is an open-access publication distributed under the terms of the Creative Commons Attribution license. 

rate, temperature, and blood pressure, which was controlled by the simulation software. A technician from our simulation center operated the simulation mannequin. Stethoscopes were available for examination. We provided copies of lab results to the participants on request (Appendices B and D).

### Personnel

One to two facilitators were required to perform the simulation session as well as a technician from the simulation department if the facilitators were not experienced in operating the simulation equipment. The facilitators included PGY 3-PGY 4 pediatric and MP residents, PEM fellows, and attendings. The junior facilitator played the role of the moderator and guided the flow of each case, asked questions of the learners, and provided guidance from a resident perspective. Senior facilitators provided additional guidance on decision-making and logistics, on when to involve higher-level providers, and on interattending variability in practice. Alternately, a solo facilitator (resident, fellow, or attending) could also run the session with the help of a technician to run the simulation equipment.

The PGY 1 participants served in the role of primary provider. When additional participants were present, they served in the role of additional residents working in the department from whom the primary participant could ask for assistance if needed. Participants then rotated roles in the second case.

### Implementation

At our institution, first-year pediatric and MP residents spent a total of 2-6 weeks on PED rotations divided into blocks of 2-4 weeks. During their first PED block of the academic year, interns were invited to participate in our orientation session by email. This email included the aforementioned review articles on the topics to be read prior to the session as well as a preparticipation survey (Appendix F). Sessions took place the morning of the first day of the rotation.

Notably, during the first pilot iteration of this project, we invited only half of the PGY 1 class to participate. Following positive feedback, supportive survey results, and residency administration endorsement, we expanded the invitation to all PGY 1 residents in the second year of the project.

Participating PGY 3-PGY 4 residents, fellows, and attendings volunteered to lead the sessions. In the days before their session, they received an email with the cases (Appendices A and C) as well as a comprehensive document describing the agenda of the orientation, how to run the simulation session (Appendix E), and the overall goals of the PED rotation.

On the day of the session, the facilitators set up the simulation mannequin and its electronic vitals board in either the resuscitation bay or an available patient room within the ED, with the help of a simulation technician. On the participants' arrival, the facilitators gave them an overview of simulation-based learning and the objectives of the session. The facilitators provided the first participant with the chief complaint and age of the child and instructed the participant to enter the patient room to begin the encounter. We asked the residents to interact in the room as they would within a normal patient encounter: talking to the patient, parent, and/or nurse; examining the patient; and ordering any desired labs, imaging, or treatments. We provided lab results in paper form when requested by the participant (Appendices B and D) and offered radiograph impressions verbally. Each case generally ran 15-20 minutes. After pursuing the encounter to the point of patient stabilization and completion of the initial workup, the case was ended, and the facilitators guided participants through the debriefing questions and teaching points (Appendix G). This process was then repeated for the second simulation case. After both cases were complete, the facilitators discussed ED-specific strategies on how to manage the ED workflow and unique challenges faced in the PED. The sessions in total ran 60-75 minutes. Facilitators could choose to distribute teaching evaluations for themselves at the end of the session.

### Assessment

For intra-activity assessments, we created critical actions checklists for each case to reflect management choices that would be expected at the level of PGY 1 (Appendix H). The debriefing materials combined with the checklist addressed the expected medical and practical management of the cases, including when a patient should be staffed with a fellow or attending, whether the patient should be moved to the resuscitation bay, and which labs, imaging, or medications were indicated. These assessments were not recorded but were simply used for individualized feedback within the sessions.

We also sought to evaluate the efficacy of our orientation in improving learner self-confidence in decision-making and independent management. Our implementation the first year was constructed as a feasibility pilot, with only half of the PGY 1 pediatric and MP residents participating in the simulation orientation while the others received the standard orientation materials. The trainees were randomized into one of the two groups based on scheduling convenience. An online survey was distributed immediately prior to the rotation, immediately after the orientation curriculum (intervention group only), and 1 week after the trainees' last PED shift of the block. Using Likert

Copyright © 2020 Holzemer et al. This is an open-access publication distributed under the terms of the Creative Commons Attribution license.

scales, we asked residents to rate their level of self-confidence in independent decision-making, stabilization of acutely ill patients, and initiating treatment prior to staffing, plus whether they felt the in-person orientation was helpful. Survey questions are included in Appendix F. We compiled anonymized data, reporting them as mean Likert scores with standard deviations, and used independent *t* tests for statistical analyses.

During the second year of implementation, we invited all PGY 1 pediatric and MP residents to participate. We again distributed the same online survey immediately prior to the rotation and 1 week after the residents' last ED shift of the block. During this iteration, we linked the results of each individual resident's pre- and postrotation surveys to allow for paired data analyses. We compiled anonymized data and reported them as mean Likert scores with standard deviations and mean change in scores. We used paired *t* tests for statistical analyses.

The Institutional Review Board at the University of Michigan approved this study with exemption status as research on the effectiveness of or the comparison among instructional techniques, curricula, or classroom management methods (HUM00146625). We performed all statistical analyses using RStudio Version 1.1.463.

### Debriefing

We provided the facilitators with guidelines for structured debriefing (Appendix G) after each case. This allowed for both formal and informal elements in the debrief, which was designed to maximize the interns' education and focus on their particular questions/concerns about each scenario. We focused our debriefing on medical knowledge/patient assessment, learner self-reflection, and general ED patient care points. We highlighted specific medical information that we wanted the learners to take away from each case. Learner self-reflection focused on situational awareness/department management issues, asking them to consider whether they would defer to a senior resident in the PED to pick up the sicker patients, when they would ask a senior physician for help, and what type of workup they felt comfortable initiating independent of the senior physician. Finally, we gave the interns an opportunity to ask any questions about working in the PED. Commonly reviewed topics included documentation, workflow, and attendings' expectations of residents.

During the first year of this curriculum, we found that learners were enthusiastic about the debriefing portion, so we decided to extend the time of the overall session to accommodate a longer debrief.

## Results

During the pilot year, 13 PGY 1 pediatric and MP residents participated in the sessions in groups of one to three. The sessions were each generally run by two of the authors. After the first year's success, we edited the cases to make it easier for others to run the sessions and subsequently invited all PGY 1 pediatric and MP residents to participate the following year. Twenty-six participants completed the sessions in the second year of implementation. As shown below in more detail, the cases were well received, with very positive feedback from participants, resident instructors, and attending/fellow facilitators. Overall, seven attending/fellows and 10 senior residents used the curriculum to lead at least one session for these participants.

### First Year

During our first pilot year, a total of 13 PGY 1 residents participated in the orientation sessions. Within this intervention group, eight (62%) prerotation surveys, seven (53%) postorientation survey, and seven (53%) postrotation surveys were completed. An additional 15 residents were sent surveys as part of the control group, and of these, 10 (67%) prerotation surveys and 11 (73%) postrotation surveys were completed. Responses are summarized in Table 1. In the intervention group, there was a significant difference in confidence in independent

**Table 1.** PGY 1 Resident Survey Responses to the Pilot Year of Orientation Sessions

| | Control Group | | | Intervention Group | | | |
|---|---|---|---|---|---|---|---|
| **Item** | **Prerotation ($n$ = 10): $M \pm SD$** | **Postrotation ($n$ = 11): $M \pm SD$** | ***p*** | **Preorientation ($n$ = 8): $M \pm SD$** | **Postorientation ($n$ = 7): $M \pm SD$** | **Postrotation ($n$ = 7): $M \pm SD$** | ***p*** |
| Confidence in independent decision-making[a] | 2.2 ± 1.2 | 2.5 ± 0.7 | .45 | 1.9 ± 0.4 | 2.4 ± 0.5 | 2.6 ± 0.5 | .01[c] |
| Preparedness to rapidly assess and stabilize unstable patients[b] | 1.6 ± 0.7 | 1.9 ± 0.7 | .33 | 1.4 ± 0.5 | 2.1 ± 0.7 | 2.1 ± 0.7 | .03[c] |
| Comfort initiating treatment of a patient before staffing[a] | 2.1 ± 1.2 | 2.4 ± 0.7 | .55 | 1.5 ± 0.8 | 2.3 ± 0.5 | 1.9 ± 0.4 | .26 |

[a]Rated on a 5-point Likert scale (1 = lowest, 5 = highest).
[b]Rated on a 4-point Likert scale (1 = lowest, 4 = highest).
[c]Denotes significance of $p < .05$.

Copyright © 2020 Holzemer et al. This is an open-access publication distributed under the terms of the Creative Commons Attribution license.

**Table 2.** PGY 1 Resident Survey Responses Following the Second Year of Orientation Sessions

| Item | Preorientation ($n$ = 10): $M \pm SD$ | Postrotation ($n$ = 11): $M \pm SD$ | Mean Difference | $p$ |
|---|---|---|---|---|
| Confidence in independent decision-making[a] | 1.9 ± 0.6 | 2.6 ± 0.7 | 0.7 | .007[c] |
| Preparedness to rapidly assess and stabilize unstable patients[b] | 1.6 ± 0.5 | 2.2 ± 0.7 | 0.6 | .01[c] |
| Comfort initiating treatment of a patient before staffing[a] | 1.7 ± 0.6 | 2.4 ± 0.9 | 0.8 | .006[c] |

[a]Rated on a 5-point Likert scale (1 = lowest, 5 = highest).
[b]Rated on a 4-point Likert scale (1 = lowest, 4 = highest).
[c]Denotes significance of $p < .05$.

decision-making across the surveys ($p = .01$). A significant increase in confidence was seen when comparing before and after the orientation ($p = .04$) and also seen when comparing preorientation to postrotation ($p = .01$). No difference was found between postorientation and postrotation scores ($p = .63$).

The intervention group showed a similar pattern of significant differences in self-reported preparedness to assess and stabilize unstable patients across surveys ($p = .03$). There was a significant increase in preparedness with both postorientation ($p = .03$) and postrotation ($p = .03$) surveys compared with preorientation surveys. There was no difference between the postorientation and postrotation surveys ($p = 1.00$).

Thus, the improvements in confidence in decision-making and preparedness to assess and stabilize unstable patients appeared after orientation and continued through the rotation. There were no significant differences in the control group across the surveys on these two questions ($p > .05$). Comfort in initiating treatment of a patient before staffing did not show significant differences for either the intervention group or the control group ($p > .05$).

Second Year

In our second year of implementation, 26 interns participated, 16 of whom completed both the preorientation and postrotation surveys. The results of paired *t*-test analyses on the linked responses are summarized in Table 2. This group similarly showed a significant increase in confidence in independent decision-making (mean increase = 0.7, $p = .007$) as well as in self-reported preparedness to assess and stabilize unstable patients (mean increase = 0.6, $p = .01$). The group also showed significantly increased comfort in initiating treatment prior to staffing with a fellow or attending (mean increase = 0.8, $p = .006$).

The combined data from both years of participating interns showed that 35% found the orientation very helpful, 40% found it moderately helpful, and 25% found it somewhat helpful. None felt it was not at all helpful in improving their care during their PED rotation.

## Discussion

Our experience shows that this curriculum could successfully be implemented as part of a residency training program. We established this orientation curriculum in order to improve resident self-confidence and sense of preparedness in the PED. This can be a stressful environment for new providers due to its fast pace and high variability in patient acuity. Moreover, patient workflow and supervision of trainees in the PED are very different than on inpatient wards or outpatient clinics. We thus feel that we have added a novel curriculum to the existing curricula available in *MedEdPORTAL* as ours focuses explicitly on the logistics, decision-making, and workflow of resident-based care in the ED setting.

Utilizing near-peer teaching, this curriculum also helped senior residents hone their teaching skills, including using simulation as a teaching tool, guiding higher-level decision-making, and adapting sessions to various learner needs and experiences.

Our targeted goals of improvement in resident self-confidence and preparedness can be difficult to quantify given that they are subjective and self-reported. However, we felt that comparing an intervention group to a concurrent control group was our best option for assessing the new curriculum while keeping the process feasible within our overall training program. The fact that we saw an improvement in many of our targeted markers in the intervention group immediately after orientation and at the end of the rotation that was not seen in the control group supports the efficacy of our project. We understand that with such small sample sizes and numerous likely confounding factors such as individual baseline knowledge and prior ED experience, these findings are in no way definitive. However, our results were compelling enough that both the residency program leadership and the division of PEM committed to adding our orientation to the residency curriculum and to providing facilitators on a regular basis.

With this departmental support, we felt our class-wide implementation was successful as demonstrated by significant

Copyright © 2020 Holzemer et al. This is an open-access publication distributed under the terms of the Creative Commons Attribution license.

improvement seen again in our target outcomes of self-confidence in decision-making, sense of preparedness to assess and stabilize unstable patients, and comfort initiating treatment prior to staffing a patient. While the mean differences were modest (0.6-0.8 increase on the respective Likert scales), it should be noted that they were achieved after a 1-hour orientation and approximately seven 8-hour shifts during the residents' first rotation block.

We believe our PED orientation and its assessment contribute to the body of similar literature examining the value of simulation-based orientations to boost trainee self-confidence in patient management such as those shown in pediatric critical care,[7] surgery,[5] and obstetrics training programs.[6] We also believe that the curriculum could be extended to other levels of learners, including medical students as well as more advanced learners, such as senior residents or fellows, by adjusting the critical actions checklist to the trainee's expected level of practice.

The biggest barrier to implementation of our curriculum was difficulty in scheduling the sessions. We found that the sessions ran best with two or three interns per group. Given that we had 32 first-year residents, this meant recruiting facilitators for a minimum of 11 sessions. Variables including the size of the target group at an institution, inconsistent intern scheduling throughout the academic year, and staggered shift start times can make it a challenge to schedule these sessions. Despite these barriers, over 80% of the class was able to participate over the course of our second year. While, in its current form, the curriuclum is meant to orient learners as they begin their work in an ED setting, it could also likely be incorporated into a more general orientation curriculum for a residency program in the form of a boot camp.

Responding to learner feedback, we may try to incorporate medical record/ordering experience into future sessions. We are also planning to expand our discussions on general intern expectations for the rotation.

We acknowledge that we did not use competence-focused measures. Previous studies have called into question the correlation between self-reported confidence and actual competence.[15] The data analyses of our initial iteration were also limited by the fact that our group sizes were small, and our randomization and analyses did not control for important resident characteristics such as previous experience in an emergency room or career interest.

Despite these limitations, our case-based orientation curriculum utilizing simulation mannequins was very well received, and we feel that it can improve pediatric and MP PGY 1 residents' self-confidence and sense of preparedness during their first rotation in the PED.

## Appendices

A. Case 1 Status Asthmaticus.docx
B. Lab Handout Status Asthmaticus.docx
C. Case 2 Sepsis.docx
D. Lab Handout Sepsis Case.docx
E. Case Instructions for Facilitators.docx
F. Participant Surveys.docx
G. Debriefing Tools and Teaching Points.docx
H. Critical Actions Checklist.docx

*All appendices are peer reviewed as integral parts of the Original Publication.*

**Nicholas F. Holzemer, MD:** Resident, Internal Medicine-Pediatrics, Michigan Medicine

**Elaine S. Pomeranz, MD:** Associate Professor, Department of Emergency Medicine, Michigan Medicine; Associate Professor, Department of Pediatrics, Michigan Medicine

**Sarah Tomlinson, MD:** Assistant Professor, Department of Emergency Medicine, Michigan Medicine; Assistant Professor, Department of Pediatrics, Michigan Medicine

**Disclosures**
None to report.

**Funding/Support**
None to report.

**Prior Presentations**

Holzemer N, Tomlinson S. Residents report low self-confidence in independent decision making and patient stabilization in the pediatric emergency department. Poster presented at: University of Michigan Pediatric Research Symposium; November 13-14, 2017; Ann Arbor, MI.

Holzemer N, Hemberg J, Pomeranz E, Tomlinson S. Piloting a new simulation-based orientation to improve emergency medicine rotations in pediatric residency. Poster presented at: Pediatric Academic Societies Meeting; May 5, 2018; Toronto, Ontario, Canada.

Holzemer N, Tomlinson S. Simulation-based peer-led orientation improves intern confidence and preparedness. Poster presented at: University of Michigan Pediatric Research Symposium; May 13, 2019; Ann Arbor, MI.

Copyright © 2020 Holzemer et al. This is an open-access publication distributed under the terms of the Creative Commons Attribution license.

### Ethical Approval

The University of Michigan Institutional Review Board approved this study.

## References

1. Chen EH, Cho CS, Shofer FS, Mills AM, Baren JM. Resident exposure to critical patients in a pediatric emergency department. *Pediatr Emerg Care*. 2007;23(11):774-778. https://doi.org/10.1097/PEC.0b013e318159ffef
2. Mittiga MR, Schwartz HP, Iyer SB, Gonzalez del Rey JA. Pediatric emergency medicine residency experience: requirements versus reality. *J Grad Med Educ*. 2010;2(4):571-576. https://doi.org/10.4300/JGME-D-10-00106.1
3. Fernandez GL, Page DW, Coe NP, et al. Boot camp: educational outcomes after 4 successive years of preparatory simulation-based training at onset of internship. *J Surg Educ*. 2012;69(2):242-248. https://doi.org/10.1016/j.jsurg.2011.08.007
4. Jeffers JM, Poling S. The development and implementation of a 12-month simulation-based learning curriculum for pediatric emergency medicine fellows utilizing debriefing with good judgment and rapid cycle deliberate practice. *BMC Med Educ*. 2019;19:22. https://doi.org/10.1186/s12909-018-1417-6
5. Rialon KL, Barfield ME, Elfenbein DM, Lunsford KE, Tracy ET, Migaly J. Resident designed intern orientation to address the new ACGME Common Program Requirements for resident supervision. *J Surg Educ*. 2013;70(3):350-356. https://doi.org/10.1016/j.jsurg.2012.11.015
6. Hiraoka M, Kamikawa G, McCartin R, Kaneshiro B. A pilot structured resident orientation curriculum improves the confidence of incoming first-year obstetrics and gynecology residents. *Hawaii J Med Public Health*. 2013;72(11):387-390.
7. Nishisaki A, Hales R, Biagas K, et al. A multi-institutional high-fidelity simulation "boot camp" orientation and training program for first year pediatric critical care fellows. *Pediatr Crit Care Med*. 2009;10(2):157-162. https://doi.org/10.1097/PCC.0b013e3181956d29
8. Reid J, Stone K. Pediatric Emergency Medicine Simulation Curriculum: status asthmaticus. *MedEdPORTAL*. 2014;10:9660. https://doi.org/10.15766/mep_2374-8265.9660
9. Reid J, Stone K. Pediatric Emergency Medicine Simulation Curriculum: septic shock. *MedEdPORTAL*. 2013;9;9639. https://doi.org/10.15766/mep_2374-8265.9639
10. Sagalowsky S, Boyle T, Winn A, et al. Four core cases: a simulation curriculum for pediatrics residents. *MedEdPORTAL*.2014;10:9943. https://doi.org/10.15766/mep_2374-8265.9943
11. Rideout M, Raszka W. Hypovolemic shock in a child: a pediatric simulation case. *MedEdPORTAL*. 2018;14:10694. https://doi.org/10.15766/mep_2374-8265.10694
12. Frallicciardi A, Nowicki T, Herbst M, Flores A. A simulation-based emergency medicine PGY-1 resident orientation curriculum. *MedEdPORTAL*. 2015;11;10115. https://doi.org/10.15766/mep_2374-8265.10115
13. Jones BP, Fleming GM, Otillio JK, Asokan I, Arnold DH. Pediatric acute asthma exacerbations: evaluation and management from emergency department to intensive care unit. *J Asthma*. 2016;53(6):607-617. https://doi.org/10.3109/02770903.2015.1067323
14. Mendelson J. Emergency department management of pediatric shock. *Emerg Med Clin North Am*. 2018;36(2):427-440. https://doi.org/10.1016/j.emc.2017.12.010
15. Barnsley L, Lyon PM, Ralston SJ, et al. Clinical skills in junior medical officers: a comparison of self-reported confidence and observed competence. *Med Educ*. 2004;38(4):358-367. https://doi.org/10.1046/j.1365-2923.2004.01773.x

**Received:** July 6, 2019
**Accepted:** December 15, 2019
**Published:** June 30, 2020

Copyright © 2020 Holzemer et al. This is an open-access publication distributed under the terms of the Creative Commons Attribution license.
